# Molecular epidemiology and characterization of human coronavirus in Thailand, 2012–2013

**DOI:** 10.1186/s40064-016-3101-9

**Published:** 2016-08-26

**Authors:** Rapeepun Soonnarong, Ilada Thongpan, Sunchai Payungporn, Chanpim Vuthitanachot, Viboonsuk Vuthitanachot, Preeyaporn Vichiwattana, Sompong Vongpunsawad, Yong Poovorawan

**Affiliations:** 1 Department of Biochemistry, Faculty of Medicine, Chulalongkorn University, Bangkok, Thailand; 2 Center of Excellence in Clinical Virology, Department of Pediatrics, Faculty of Medicine, Chulalongkorn University, Bangkok, Thailand; 3 Chum Phae Hospital, Khon Kaen, Thailand

**Keywords:** Human coronavirus (HCoV), Epidemiology, Respiratory tract infection (RTI), Thailand

## Abstract

**Background:**

Coronavirus causes respiratory infections in humans. To determine the prevalence of human coronavirus (HCoV) infection among patients with influenza-like illness, 5833 clinical samples from nasopharyngeal swabs and aspirates collected between January 2012 and December 2013 were examined.

**Results:**

HCoV was found in 46 (0.79 %) samples. There were 19 (0.32 %) HCoV-HKU1, 19 (0.32 %) HCoV-NL63, 5 (0.09 %) HCoV-229E, and 3 (0.05 %) HCoV-OC43. None of the sample tested positive for MERS-CoV. The majority (54 %) of the HCoV-positive patients were between the ages of 0 and 5 years. HCoV was detected throughout the 2-year period and generally peaked from May to October, which coincided with the rainy season. Phylogenetic trees based on the alignment of the spike (*S*) gene sequences suggest an emergence of a new clade for HCoV-229E.

**Conclusions:**

The data in this study provide an insight into the prevalence of the recent circulating HCoVs in the region.

## Background

Human coronavirus (HCoV) is an enveloped positive-strand RNA virus with ~27–32 kb genome. Its genome contains five major open reading frames (Orfs) encoding the replicase polyproteins (Orf 1a and Orf 1b), spike (S), envelope (E), membrane (M), and nucleocapsid (N) proteins (Hilgenfeld and Peiris 2013). HCoV belongs to the order *Nidovirales*, family *Coronaviridae* and the genus *Coronavirus* (Lai and Cavanagh 1997). Virions are pleomorphic with diameters between 60 and 220 nm. There are four genera of coronaviruses (*Alpha*-, *Beta*-, *Gamma*- and *Deltacoronavirus*) based on serologic and phylogenetic characterization. Among the five recognized human coronaviruses, HCoV strains 229E and NL63 are alphacoronaviruses, while HCoV strains OC43, HKU1, and SARS (responsible for the severe acute respiratory syndrome) are betacoronaviruses (Gaunt et al. 2010; Gonzalez et al. 2003; Drosten et al. 2003). In addition, a novel genotype of coronavirus was identified in Saudi Arabia in 2012, later referred to as MERS-CoV (for the Middle East respiratory syndrome coronavirus), which was isolated from the sputum of a 60-year-old man in Saudi Arabia who presented acute pneumonia and renal failure with a fatal outcome (Hilgenfeld and Peiris 2013; Zaki et al. 2012). It was subsequently found to be most closely related to the bat betacoronavirus HKU4 and HKU5 (Gonzalez et al. 2003). Fortunately, the conserved structure and function of the polymerase made it possible to develop a PCR assay based on the polymerase gene region to accurately differentiate new coronaviruses as was done with MERS-CoV and other novel coronaviruses (Rota et al. 2003; Moës et al. 2005).

HCoVs are recognized as one of the most frequent causes of upper respiratory tract infection in elderly adults leading to acute pneumonia and renal failure (Memish et al. 2013; Gorse et al. 2009). HCoV infection is thought to participate in the exacerbations of chronic obstructive pulmonary disease, congestive heart failure, and other chronic diseases necessitating emergency care and long-term hospitalization. In addition to respiratory syncytial virus, parainfluenza virus, adenovirus, and influenza virus, HCoVs are also known to cause the common cold especially among children (Moës et al. 2005; Lu et al. 2012). Furthermore, HCoVs are associated with mild to severe upper and lower respiratory tract illness and can cause more serious respiratory diseases in children, the elderly and people with underlying conditions (McIntosh et al. 1974; van Elden et al. 2004).

The study of coronaviruses has sometimes been difficult due to limitations in cell culture and serology. Thus, epidemiological and viral prevalence data are valuable in investigating the emergence of HCoV infection. Using reverse-transcription polymerase chain reaction (RT-PCR) and phylogenetic analysis, we characterized HCoVs identified in Thai patients with respiratory tract infection between 2012 and 2013.

## Methods

### Clinical samples

This study was approved by the Faculty of Medicine of Chulalongkorn University (IRB 388/56). A total of 5833 clinical samples from patients with influenza-like illness were obtained between January 2012 and December 2013 for routine testing of respiratory viruses as part of an epidemiological surveillance (Auksornkitti et al. 2014; Prachayangprecha et al. 2013; Sriwanna et al. 2013). From these, 5196 nasopharyngeal swabs (NPS) were collected from patients with upper respiratory tract infections who sought treatment at the Bangpakok 9 International Hospital and Chum Phae Hospital (Khon Kaen, Thailand). Additionally, 637 nasopharyngeal aspirates (NPA) were collected from patients with lower respiratory tract infections who were admitted to King Chulalongkorn Memorial Hospital and Chon Buri Hospital. All samples were stored in the viral transport medium (prepared according to The World Health Organization guideline). Patient identifiers were removed and all samples were anonymous, although data on demographics, symptoms, history of illness, results of clinical examination and laboratory investigations were retained for analysis.

### Coronavirus detection

Viral DNA/RNA was extracted using HiYield Viral Nucleic Acid Extraction Kit (RBC Bioscience, Taipei, Taiwan, ROC). Complementary DNA was synthesized with random hexameric primers and ImProm-II (Promega, Madison, WI, USA) according to the manufacturer’s instructions. HCoV was identified using the semi-nested RT-PCR to amplify the RNA-dependent RNA polymerase (*RdRp*) gene of HCoV and the *N* gene of MERS-CoV as previously described (Corman et al. 2012; Kon et al. 2012). For controls, a panel of viral nucleic acid extracted from samples previously tested positive for influenza A virus [subtype H1N1 (pandemic 2009), H3N2], influenza B virus, respiratory syncytial virus (RSV), human parainfluenza virus (HPIV) and human adenovirus was used to determine the specificity of the HCoV semi-nested RT-PCR. PCR was performed using the Perfect*Taq*^TM^ Plus Master Mix kit (5 PRIME, Hamburg, Germany) and primers (Table 1) in 25 µL reaction volume under the following conditions: initial denaturation at 94 °C for 3 min, 40 cycles of 94 °C for 30 s, 55–58 °C for 30 s, 72 °C for 40 s, and a final extension at 72 °C for 7 min. Expected amplicons for RdRp and N were ~450 and ~260 bp, respectively. All the specimens were positive for GAPDH, which served as an internal control.

**Table 1:** Sequences of primers used to identify HCoVs in this study

Gene	Primer	Genotype	Sequence (5′–3′)^a^	PCR application
*RdRp*	SP6-CoV_16053_F		ATTTAGGTGACACTATAGGGTTGGGAY TAYCCTAARTGTGA	First and second round
	CoV-16594_R		TAYTATCARAAYAATGTCTTTATGTC	First round
	CoV-Pan_16510_R		TGATGATGGNGTTGTBTGYTATAA	Second round
*S*	HCoV-S229E_F1	229E	GTGGGTGCACTACCTAAGAC	First round
	HCoV-S229E_R1	229E	CGTGGTTGAACAGCAATTATAGAACC	
	HCoV-S229E_F2	229E	GAGTTTGTTATTTCACGCACAGGAC	Second round
	HCoV-S229E_R2	229E	CCATCTGCACAAACGCCAAAAC	
	HCoV-SHKU1_F1	HKU1	TCACCTCTTAATTGGGAACGTA	First round
	HCoV-SHKU1_R1	HKU1	CATTAGAACAAGTGGTGCCAC	
	HCoV-SHKU1_F2	HKU1	GATTTGCAGTTGGGCAGTTCTGG	Second round
	HCoV-SHKU1_R2	HKU1	AAAGGCATCAGGACTACAAA	
	HCoV-SNL63_F1	NL63	GACACCACAATACCTTTTGG	First round
	HCoV-SNL63_R1	NL63	CTGGTTGGTTACATGGTGTCAC	
	HCoV-SNL63_F2	NL63	CATGTTAGCACTTTTGTGGGT	Second round
	HCoV-SNL63_R2	NL63	CCACCAGCAAGTGACTGGTTTG	
	HCoV-SOC43_F1	OC43	GTCGGTGCCCTCTCCATTAAATT	First round
	HCoV-SOC43_R1	OC43	GGCCGCAGAAACACGAC	
	HCoV-SOC43_F2	OC43	AATATGAGCAGCCTGATGTC	Second round
	HCoV-SOC43_R2	OC43	CCGAAATAGCAATGCTGGTTC	
*N*	NSeq-Fwd	MERS	CCTTCGGTACAGTGGAGCCA	First round
	NSeq-Fnest	MERS	TGACCCAAAGAATCCCAACTAC	Second round
	NSeq-Rev	MERS	GATGGGGTTGCCAAACACAAAC	First and second round

^a^ Y = (C/T) and R = (A/G)

To identify the types of HCoVs (HCoV-229E, HCoV-NL63, HCoV-OC43, and HCoV-HKU1), the *S* gene was genotyped using nested RT-PCR. Amplification condition was the same as above except that the annealing temperature was 50 °C. Amplicons were agarose gel-purified using the Expin Gel SV kit (GeneAll, Seoul, Korea) and DNA sequencing was performed by First BASE Laboratories (Seri Kembangan, Selangor, Malaysia).

### Sensitivity of semi-nested RT-PCR for coronavirus detection

PCR products from the amplification of HCoV-229E, HCoV-NL63, HCoV-OC43, HCoV-HKU1 and MERS-CoV were cloned into pGEM-T Easy Vector System (Promega, CA, USA) according to the manufacturer’s instructions. RNA transcripts from these five coronaviruses were used as standards to validate assay sensitivity. The sensitivity of the semi-nested RT-PCR assay was established for each coronavirus by testing transcripts of known concentrations from serial dilution.

### Sequence and phylogenetic analysis

Nucleotide sequences of the *RdRp* and *S* genes were edited using Chromas Lite (version 2.1.1) and compared to the HCoV reference strains available in GenBank using the Basic Local Alignment Search Tool (BLAST) program (www.ncbi.nlm.nih.gov/Blast.cig). Multiple alignments of the nucleotide sequences utilized Clustal W in BioEdit (version 7.0.9). Phylogenetic trees were constructed using the neighbor-joining method and the Kimura two-parameter distance model (MEGA version 6.06) and evaluated by 1000 bootstrap pseudo-replicates.

### Nucleotide sequences

The nucleotide sequences of the *RdRp* gene (accession numbers KJ866056–KJ866101) and the *S* gene (accession numbers KJ866102–KJ866147) identified in this study were deposited in the GenBank database.

## Results

### Sensitivity and specificity of semi-nested RT-PCR assays for coronavirus detection

Serial dilutions of coronavirus RNA transcripts (HCoV-229E, HCoV-NL63, HCoV-OC43, HCoV-HKU1, and MERS-CoV) were tested using semi-nested RT-PCR. RNA transcripts for all virus types were detectable at ≤10 copies per reaction, or <400 copies/mL of sample. To evaluate detection specificity and possible cross-reactivity, nucleic acid from 18 different types of respiratory viruses were tested. All assay results were negative, and no false positive was observed.

### Prevalence of HCoV infection

Among the 5833 samples analyzed, 637 (10.9 %) were positive for influenza A virus, 206 (3.5 %) were positive for influenza B virus, 201 (3.4 %) were positive for respiratory syncytial virus A, 91 (1.6 %) were positive for respiratory syncytial virus B, and 78 (1.3 %) were positive for adenovirus (Table 2). More importantly, 46 samples (0.79 %) tested positive for HCoV. Co-infection with other respiratory viruses was not observed in the HCoV-positive samples. All samples were negative when tested for MERS-CoV *N* gene.

**Table 2:** Identification of viruses in the samples obtained from 5833 patients hospitalized for acute respiratory tract infection

Viruses	Number of samples tested positive (%)
HCoV-229E	5 (0.09)
HCoV-OC43	3 (0.05)
HCoV-NL63	19 (0.32)
HCoV-HKU1	19 (0.32)
Influenza A virus	637 (10.9)
Influenza B virus	206 (3.5)
Adenovirus	78 (1.3)
Respiratory syncytial virus A	201 (3.4)
Respiratory syncytial virus B	91 (1.6)

**Fig. 1:**
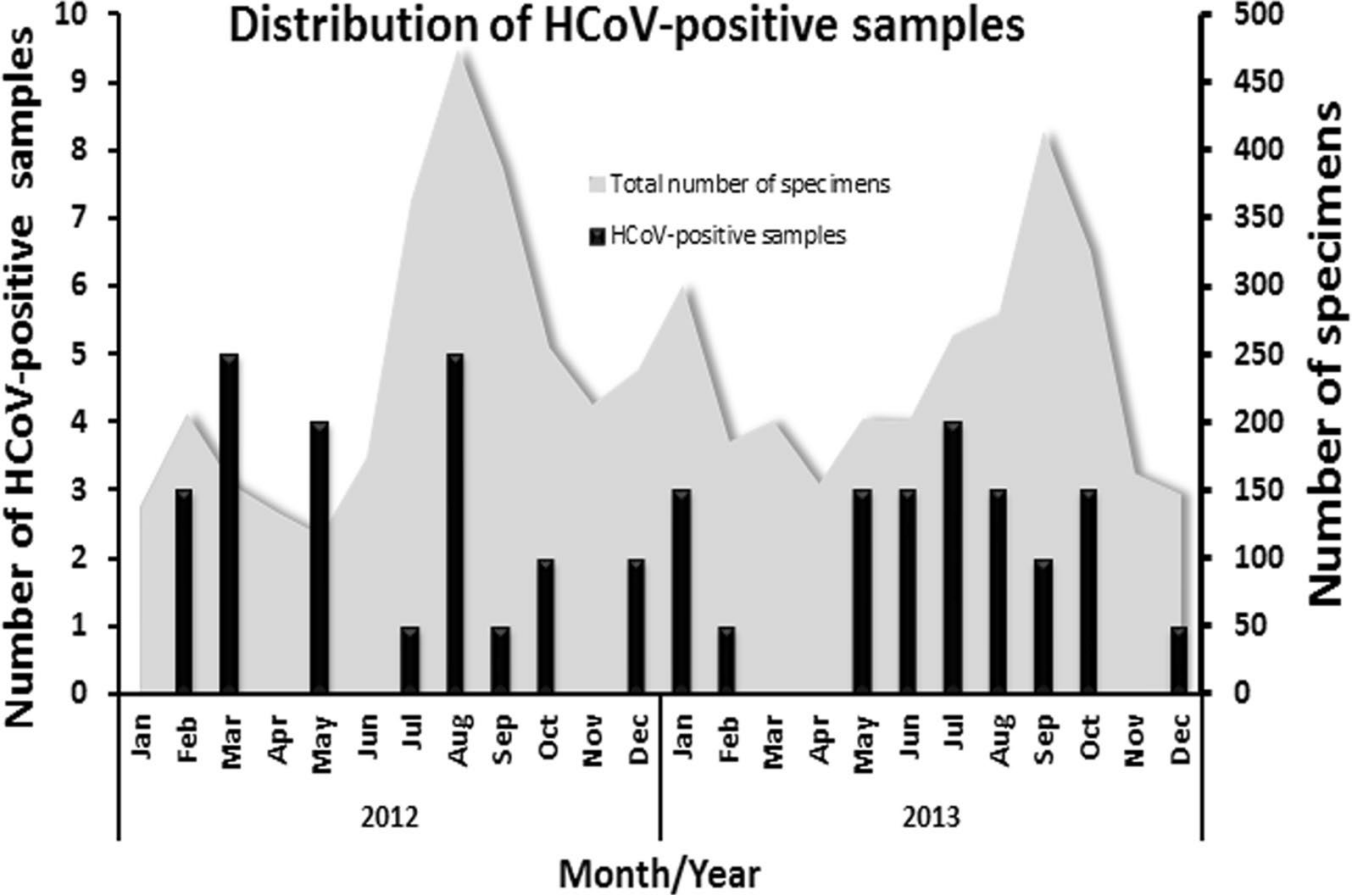
Seasonal distribution of HCoV infection from January 2012 to December 2013. *Gray area* represents the total number of specimens from influenza-like illness each month (*right scale*). *Bars* represent the number of samples tested positive for HCoV (*left scale*)

Amongst the HCoV-positive samples, 56.5 % were from men and 43.5 % were from women (gender ratio 1.3:1) (Table 3). HCoV infection was detected in all age groups, and the mean age of HCoV-infected patients was 21.37 years (min. = 4 months, max. = 93 years, mean = 27.04 years). The percentage of HCoV infection per year was 0.81 % (23/2838) in 2012 and 0.77 % (23/2995) in 2013. There were no seasonal peaks associated with HCoV and no positive samples were identified in the typically dry months of April and November during both years (Fig. 1).

**Table 3:** Demographic characteristics of individuals with respiratory tract infection

Characteristic	Specimens	
	No. specimens	Positive HCoV (%)
Gender		
Male [n (%)]	2935 (50.3)	26 (56.5)
Female [n (%)]	2898 (49.7)	20 (43.5)
Age (years)		
Median	11	8.5
Mode	1	1
Mean (SD)	19.57 (19.9)	21.37 (27.0)
Age group		
0–5 [years (%)]	2197 (37.7)	25 (54.3)
6–10 [years (%)]	732 (12.5)	2 (4.3)
11–15 [years (%)]	538 (9.2)	6 (13.0)
16–30 [years (%)]	866 (14.8)	3 (6.5)
31–60 [years (%)]	1102 (18.9)	3 (6.5)
>60 [years (%)]	397 (6.8)	7 (15.2)
Provinces		
Bangkok [n (%)]	3292 (56.4)	17 (37.0)
Khon Kaen [n (%)]	2408 (41.3)	28 (60.9)
Chon Buri [n (%)]	132 (2.3)	1 (2.2)

Numbers in parentheses indicate the percent of positive infection from total samples

**Fig. 2:**
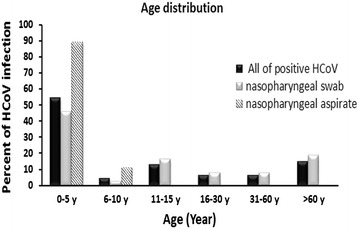
Age distribution of HCoV infection. Percentage of HCoV-positive samples were derived from different age groups (*black bars*). Positive samples tested from nasopharyngeal swab and aspirate from each age group are indicated by *gray* and *hatched bars*, respectively

The majority of HCoV (25/46 or 54 %) were detected mainly in young children between the ages of 0–5 years and 15 % in the elderly aged over 60 years (Fig. 2). Approximately 80 % of the positive samples (37/46) were isolated from the NPS samples, while the rest (9/46) were from the NPA samples. Furthermore, 46 % (17/37) of all positive NPS and 89 % (8/9) of all positive NPA samples belonged to the 0–5 year age group.

**Fig. 3:**
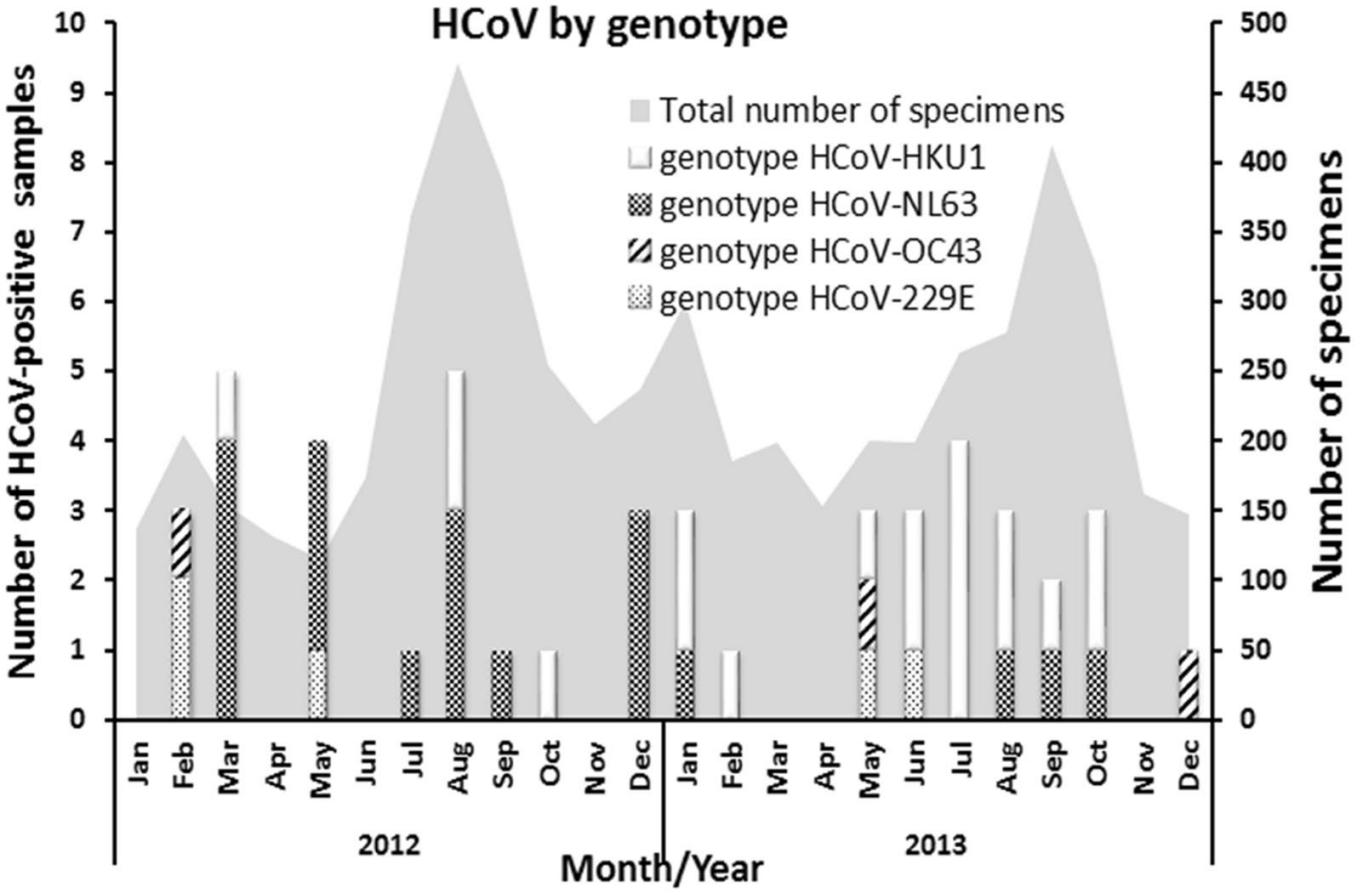
HCoV-positive samples identified by species. The total number of specimens examined is shown in *gray* (*right scale*), while the strains of HCoVs (HKU1, NL63, OC43, and 229E) identified each month are indicated as *bars* (*left scale*)

To further differentiate the 46 HCoV-positive samples, the *S* gene was sequenced. Analysis showed that these HCoV strains belonged to one of the four HCoV species (Table 2; Fig. 3). In all, 19 (0.32 %) were positive for HCoV-HKU1, 19 (0.32 %) were positive for HCoV-NL63, 5 patients (0.09 %) were positive for HCoV-229E, and 3 (0.05 %) were positive for HCoV-OC43. Relative to all HCoV-positive samples, therefore, the predominant genotypes were 41.3 % for both HCoV-HKU1 and HCoV-NL63 (19/46), followed by 11 % HCoV-229E (5/46) and 6.5 % HCoV-OC43 (3/46). Interestingly, HCoV-NL63 and HCoV-HKU1 appeared sporadically during the study period and were detected mainly in March 2012 and July 2013, respectively.

### Clinical characteristics of HCoV infections

Of the 46 HCoV-positive samples, 38 (82.6 %) were derived from patients with acute infections involving the upper respiratory tract such as the nose, sinuses, pharynx or larynx (Table 4). The remaining 8 (17.4 %) were from patients with lower respiratory tract infections. The most common clinical manifestations were fever and rhinorrhea, although several patients experienced tachypnea and hypoxemia.

**Table 4:** Clinical characteristics of patients with HCoV infection

Characteristic	HCoV-positive individuals (%)^a^			
	HCoV-229E	HCoV-OC43	HCoV-NL63	HCoV-HKU1
Total no. of patients	5	3	19	19
URTI	5	2	16 (84 %)	15 (79 %)
LRTI	0	1	3 (16 %)	4 (21 %)
Clinical symptom				
Fever	5	3	18 (95 %)	16 (84 %)
Cough	2	2	11 (58 %)	10 (53 %)
Sputum	0	1	5 (26 %)	2 (11 %)
Rhinorrhea	4	2	16 (84 %)	17 (89 %)
Vomiting	3	0	10 (53 %)	12 (63 %)
Tachypnea^b^	0	1	6 (32 %)	7 (37 %)
Hypoxemia^c^	1	1	5 (26 %)	5 (26 %)
Respiratory distress^d^	0	1	6 (32 %)	7 (37 %)
Abnormal breathing sound^e^	0	1	6 (32 %)	7 (37 %)

*URTI* upper respiratory tract infection, *LRTI* lower respiratory tract infection (pneumonia, bronchiolitis, and acute infection of the pulmonary parenchyma)

^a^Percent of HCoV-positive individuals with such symptoms

^b^Age-related definition of tachypnea by The World Health Organization for individuals <2 months (>60 breaths/min), 2–12 months (>50 breaths/min), 1–5 years (>40 breaths/min) and ≥5 years (>20 breaths/min)

^c^Oxygen saturation (SpO2) level below 95 %

^d^Including retractions (subcostal, intercostal, suprasternal), nasal flaring, and grunting

^e^Crepitation, rhonchi, and wheezing

### Molecular characterization of different HCoVs

**Fig. 4:**
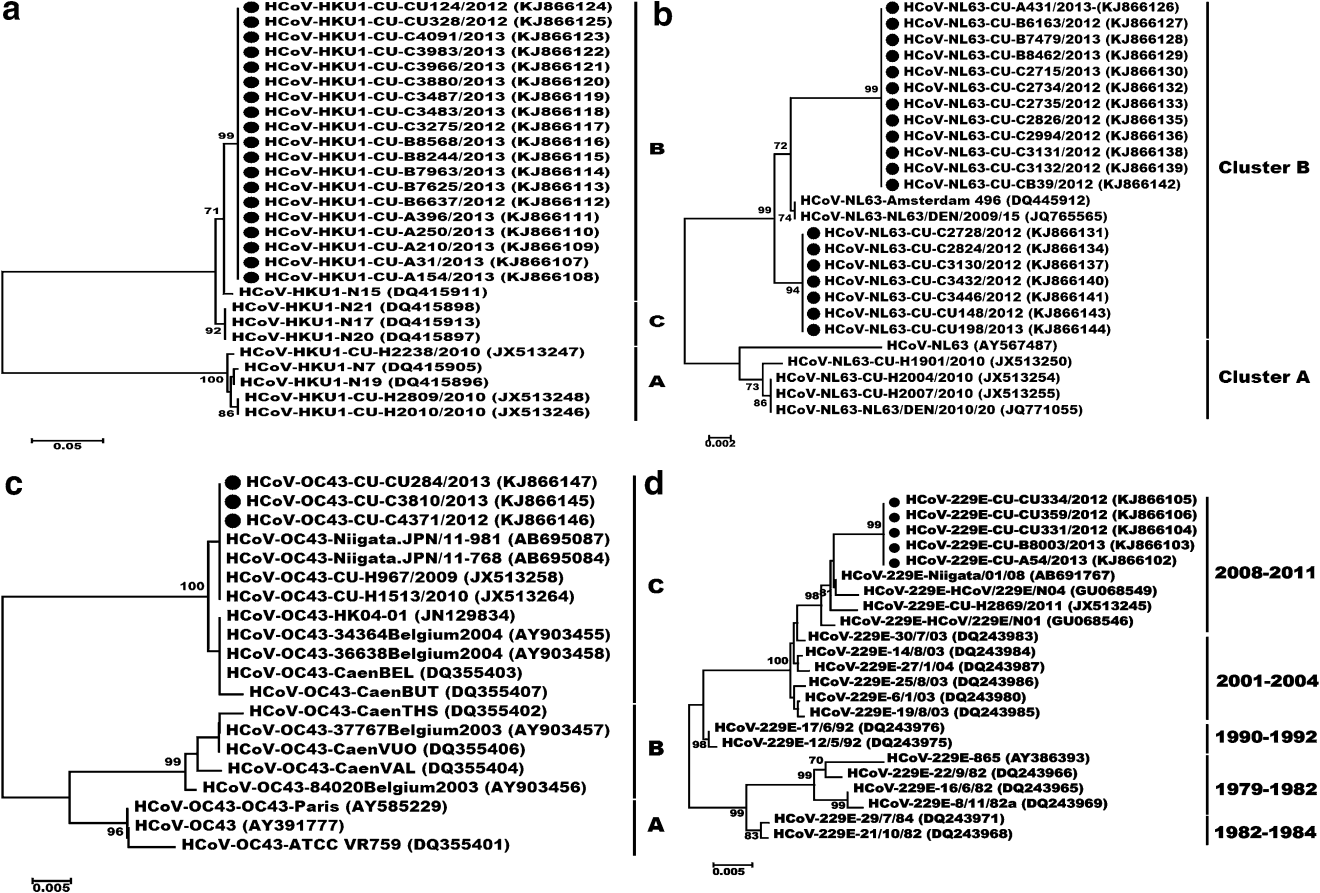
Phylogenetic analyses of the *S* genes of 4 HCoV species. Phylogenetic trees were constructed based on the partial nucleotide sequences of the *S* gene of HCoV-HKU1 (**a**), HCoV-NL63 (**b**), HCoV-OC43 (**c**), and HCoV-229E (**d**) with MEGA 6.06 software using the distance method and the neighbor-joining algorithm with Kimura 2 parameters. Phylogenetic clusters are indicated to the right of the trees. CU designation is given to each of the 46 clinical isolates examined in this study (*black dots*). The node clusters were supported by bootstrap values >70 %

To assess the relationship among the strains identified, partial nucleotide sequences of the *S* gene from HCoV-positive samples were subjected to phylogenetic analysis (Fig. 4). This region was examined as it represents the antigenic determinant of the virus and correlates with strain evolution. HCoV-HKU1 strains identified in this study formed a single cluster (~99.6 % sequence identity) within clade B together with the strain identified on mainland China. Meanwhile, HCoV-NL63 strains clustered in two different groups within the same clade. Their sequences were genetically closest to the isolates from The Netherlands and U.S.A. Three HCoV-OC43 strains grouped together with previous viruses characterized in Japan in clade C. Finally, although the 5 HCoV-229E identified in this study appeared to cluster into a new group, they demonstrated nearest genetic resemblance to strains from Japan and China.

## Discussion

Due to the awareness of MERS-CoV and other novel human coronavirus infections worldwide, this study aimed to characterize possible circulation and prevalence of HCoV in Thailand in a 2-year period. The HCoV prevalence of 0.79 % found in this study was lower than that from previous reports, which ranged from 2.1 to 5.7 % (Bellei et al. 2008; Dominguez et al. 2009; Gorse et al. 2009; Pierangeli et al. 2007). However, the rate of HCoV-positive samples found in this study was similar to the level of infection reported in countries such as mainland China (1 %) (Ren et al. 2011) and United Kingdom (0.85 %) (Gaunt et al. 2010). Several factors may account for this disparity: (1) the subjects included in this study were adult patients whereas other studies examined mainly hospitalized children (Talbot et al. 2009) or adults with underlying diseases (Dare et al. 2007), (2) the viral infection rate obtained from NPS used in this study may be lower than that of NPA (Lau et al. 2006; Talbot et al. 2009), (3) differences in the study time frame and geographical area, (4) natural varying of yearly distribution of HCoV species, and (5) differences in the techniques used for testing, such as conventional versus real-time PCR.

Although a previous study in southern Thailand found that HCoV-OC43 was the predominant strain (Suwannakarn et al. 2014), very few HCoV-OC43 infections were detected in this study. Yearly fluctuation in the predominance of HCoV strains in Thailand has been documented (Dare et al. 2007). The majority of HCoVs detected in this study was HCoV-NL63 and HCoV-HKU1. HCoV-NL63 was found mainly in 2012 and HCoV-HKU1 in 2013, while HCoV-OC43 and HCoV-229E were sporadic and less frequent.

HCoV infections elicited common symptoms such as fever, cough, headache, vomiting, muscle pain and sore throat. Upper respiratory tract infection such as rhinorrhea, sputum, and lower respiratory tract infection such as tachypnea and abnormal breath sounds were frequently observed in our patients similar to past observations (Ren et al. 2011; Dominguez et al. 2009). However, clinical presentations were slightly different among each subtype of HCoV. In this study, vomiting was more common in HCoV-229E, HCoV-NL63 and HCoV-HKU1-positive patients but not in those infected by HCoV-OC43. Lower respiratory tract infections were associated with individuals with HCoV-OC43, HCoV-NL63, and HCoV-HKU1, but not in those with HCoV-229E. Tachypnea, hypoxemia, abnormal breathing sound and signs of respiratory distress also occurred less often for HCoV-229E. These clinical features were very similar to those observed with other respiratory viruses, such as RSV, HPIV, and human metapneumovirus.

The simultaneous screening for common respiratory viruses allowed us to investigate possible co-infection of HCoV with other viral pathogens. Although parainfluenza viruses, enteroviruses, and human metapneumovirus were not tested, co-infection of HCoV with RSV was never observed (Theamboonlers et al. 2007). It may be that HCoV and other respiratory viruses do not share the same seasonal circulation. The possibility of viral coinfection with bacteria, however, cannot be ruled out. Nevertheless, confirmation of the mechanism of co-infection will require good clinical sampling and further longitudinal studies conducted over several years.

Previous characterization of the genetic variation and evolution of HCoV have utilized the whole viral genome, *S*, *RdRp*, or *N* gene. Here, the phylogenetic analysis of HCoV was performed based on the sequences of partial spike gene because the *S* gene may be more discriminating than the *RdRp* gene (Lau et al. 2006, 2011). While phylogenetic grouping of HCoV-HKU1, HCoV-NL63, and HCoV-OC43 were characterized by clades or clusters, HCoV-229E strains were typically subdivided by the year of emergence. HCoV-229E strains collectively appeared to form a new cluster similar to but distinct from previous strains in the phylogenetic tree.

## Conclusions

In summary, this study surveyed the prevalence and clinical presentations of different HCoV infections in Thai patients from 2012 to 2013. Although the numbers of HCoV-positive samples were low, we detected four species of HCoV and phylogenetically characterized their diversity. These viruses appeared to continue to cause infections globally, thus accurate and timely diagnosis will be essential. The assay described in this study can be used to assist in the rapid and accurate detection of emerging HCoV infection.

## Abbreviations

HCoV: human coronavirus
NPA: nasopharyngeal aspirate
NPS: nasopharyngeal swab
RSV: respiratory syncytial virus
RT-PCR: reverse-transcription polymerase chain reaction
